# The evolution of acute stroke care in Germany from 2019 to 2021: analysis of nation-wide administrative datasets

**DOI:** 10.1186/s42466-023-00297-x

**Published:** 2024-01-11

**Authors:** Matthias N. Ungerer, Dirk Bartig, Daniel Richter, Christos Krogias, Werner Hacke, Christoph Gumbinger

**Affiliations:** 1 Department of Neurology, University Hospital Heidelberg, Heidelberg, Germany; 2 DRG Market, Osnabrück, Germany; 3 Department of Neurology, Evangelisches Krankenhaus Herne, Academic Teaching Hospital of the Ruhr-University Bochum, Herne, Germany

**Keywords:** Thrombectomy, Health services research, Stroke

## Abstract

**Background:**

The treatment of ischemic stroke (IS) has changed considerably in recent years. Particularly the advent of mechanical thrombectomy (MTE) has revolutionized the available treatment options. Most patients in developed countries have access to intravenous thrombolysis (IVT). However access to MTE remains restricted in some regions despite efforts to increase its availability. We performed an evaluation of national datasets to monitor improvements made in access to revascularization therapies for IS patients in Germany.

**Methods:**

We analyzed national datasets on German Diagnosis-Related Groups and structured quality reports by extracting information of patients admitted with stroke with and without IVT and MTE for the period of 2019–2021. Data from 2016 and limited data for 2022 were also included for comparison.

**Results:**

Admissions with ischemic stroke declined during the years of the COVID 19 pandemic by 4.5% from 227,258 in 2019 to 216,923 in 2021. IVT rates were stable with 16.3% being treated with IVT in 2019 and 2021. MTE rates continued to increase from 7.1 to 8.4% and the number of MTE centers increased by 14.8% in the same period. Over 87.3% of MTEs were performed in centers with a case volume exceeding 50 cases per year in 2021. The largest increase in the relative share of MTEs was seen in large MTE centers (n ≥ 200). Patient age for MTEs surpassed the age for IVTs in 2019 and the proportion of patients ≥ 80 years receiving MTE continued to increase. The proportion of regions in Germany with poor MTE rates (≤ 4.1%) decreased significantly from 2019 (12.3%) to 2021 (5.3%) (*p* < 0.001).

**Conclusions:**

We found strong evidence that while IVT rates reached a temporary ceiling effect, both the absolute number of and access to MTEs continued to increase in Germany. Regional disparities have become less significant and the majority of MTEs are performed in centers with medium or high case volumes.

**Supplementary Information:**

The online version contains supplementary material available at 10.1186/s42466-023-00297-x.

## Introduction

Acute stroke treatment has changed significantly in the last decade with the establishment of dedicated stroke units (SU) and intravenous thrombolysis (IVT) as effective treatments. Their use and availability have expanded with growing scientific evidence that support their use. SU treatment and IVT rates have continued to increase in Germany in recent years and regional availability has expended as well although regional differences in access to stroke care remain [17, 18].

The recent addition of mechanical thrombectomy as a treatment for ischemic stroke (IS) has further enhanced the available options for acute stroke treatment. The possibility to effectively perform mechanical thrombectomies (MTE)s in most cases beyond the 4.5 h time window has greatly affected treatment standards and is the only available treatment option when IVT cannot be administered [7, 19]. Large MTE centers had to be established rapidly in order to provide IS patients with access to MTEs. These centers required expensive equipment and specialized medical staff in order to apply complex interventional procedures initially limiting access to MTEs to patients in urban areas. Geographical constraints continue to be an issue and result in relevant disparities in access to MTE centers. Rural patients can be significantly disadvantaged with only 27.7% of rural IS patients being treated initially in an MTE center in the period of 2016–2018 in the United States whereas 76.3% of rural patients were initially presented at centers capable of administering IVT [1, 11]. Similar differences in regional variability could be found in Germany in the period of 2016–2019 [18].

One way to increase access to MTEs has been to increase the number of centers offering MTEs. This encourages MTEs to be performed in smaller community hospitals with relatively low case volumes which can negatively affect patient outcomes [22]. Newer concepts have been developed to direct patients from rural areas to larger MTE centers. Concepts such as transfer of IS patients with large vessel occlusions to MTE centers after initial treatment with or without IVT in community hospitals (drip and ship model) or encouraging direct presentation of patients likely to have a large vessel occlusion directly to MTE centers (mothership model) have been shown to equally improve patient outcomes and have dramatically improved access to MTE for patients in developed countries [2]. Alternative approaches to send mobile neurointerventional teams to smaller MTE centers as part of a ship the doc or flying interventional team (FIT) concept have also reduced door to MTE times [8].

The developments in the capabilities and access to acute stroke care have been immense since the introduction of MTE reflected by growth in yearly treatment rates. Analysis of large databases to regularly monitor these developments are important for quality control and to identify areas where further improvements can be made.

We analyzed data from a comprehensive national administrative data registry in Germany for the period of 2019–2021 to demonstrate recent developments and trends in availability and access to IVT and MTE for the treatment of IS in this period. We set out to continue earlier analysis of the development of acute ischemic stroke care in Germany for the periods of 2010–2019 [18, 23, 24].

## Methods

We performed a retrospective cross-sectional study based on a comprehensive national administrative data registry in Germany using data on German Diagnosis-Related Groups (G-DRG) for the years 2016, 2019, 2020 and 2021.

### Data collection and study design

Data analysis has been described in detail in previous publications, upon which this follow-up study was based [16–18, 23]. In short: data is collected by the German federal Statistical Office in the destatis and InEK databases [9] on the basis of G-DRG. Data on IVT and MTE rates as well as on number of IS treated were also available for the year 2022. All other data refer to the years up to 2021. Data on structured quality reports [20] for the year 2022 will be available in 2024. Data on MTE rate by region and age distribution for 2022 will be made available in the fall of 2023 in the destatis database and could not be considered in this study. Data from 2016, which has been published previously, was included for comparison [18].

We extracted data on all cases with the main ICD-10 diagnosis of I63 (IS). Cases with suspected double inclusion of patients transferred to another hospital for acute stroke treatment or early rehabilitation were excluded from our main dataset by exclusion of “discharge key 6” (see Table 1). In-house strokes were also disregarded as only cases with the main diagnosis I63 were included. We identified all IS cases with an additional operating and procedure key (OPS code 8-020.8 and OPS code 8-836.80) to identify patients that received IVT or MTE. Data on patient sex and age were also extracted. Analysis of regional distribution of MTEs was performed by data aggregation considering 400 German administrative districts on the basis of place of residency of patients. Patients with unknown or no place of residence were excluded from the regional analysis. We defined an MTE rate of 0–4.1% as indicating poor access to MTE in our regional analysis.

**Table 1:** Exclusion of patients with multiple coding (discharge key 6) from the InEK database patient sample

Year	Cases with main ICD diagnosis of I63	Excluded cases (discharge key 6)	Cases included in our main dataset
2016	258,480	30,792	227,688
2019	257,015	29,757	227,258
2021	246,428	29,505	216,923
2022	242,491	27,012	215,479

Data on MTEs and MTE rates according to size of thrombectomy centers was extracted separately from structured quality reports as these data were not available in the destatis and InEK databases. Numbers of frequencies of MTEs varied slightly in-between data generated from the destatis and InEK databases with those generated from structured quality reports. We defined low volume (small) centers as centers with < 50 MTE cases per year on the basis of published literature [25]. Most studies have defined high volume MTE centers as having a case volume exceeding 135–150 MTE cases per year [6, 25]. We therefore defined medium sized MTE centers as having 50–199 cases per year and large volume MTE centers as having at least 200 MTE cases annually.

### Statistical analysis

Data was described using standard descriptive statistics. Percentages were rounded to one decimal place. *p* values were rounded to three decimal places. Differences in categorical variables was determined using chi squared test and t-test was used for continuous variables. Level of statistical significance was set at *p* < 0.05. SPSS Statistics (IBM Corp, IBM SPSS Statistics for Windows, Version 29.0. Armonk, NY: IBM Corp) was used for analyses.

## Results

In total, 227,258 patients were admitted with the main hospital diagnosis of ischemic stroke (IS) in 2019 to hospitals throughout Germany. This number decreased by 4.5% to 216,923 in 2021 and remained stable with 215,479 patients admitted with IS in 2022.

**Fig. 1:**
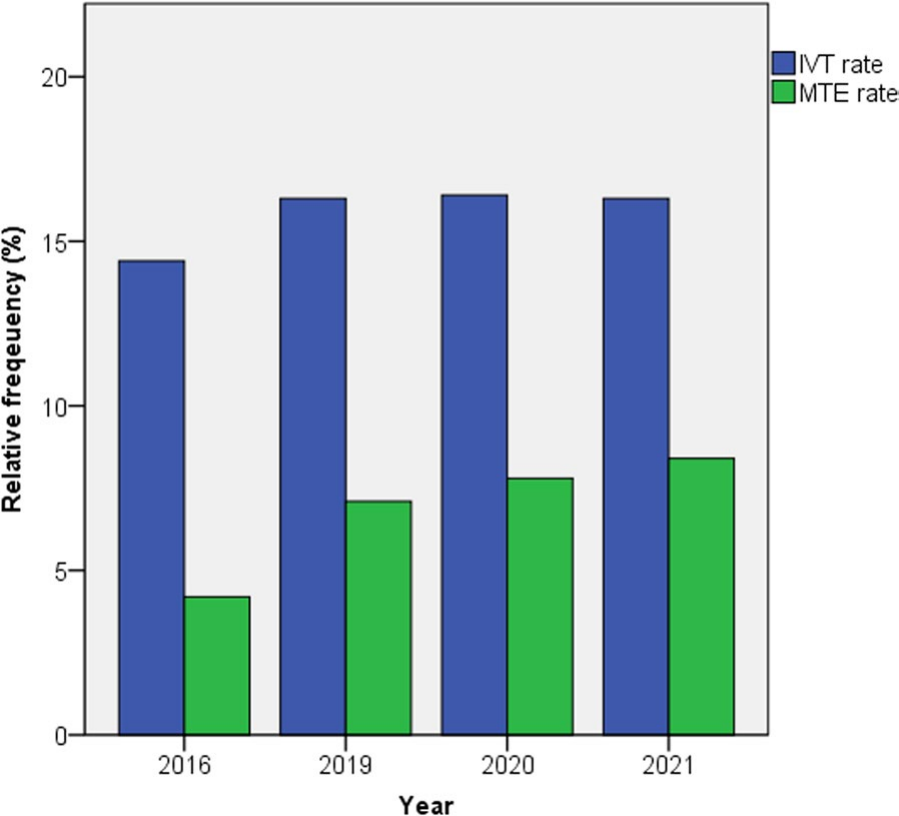
Yearly IVT and MTE rates from 2016 to 2021. Abbreviations: IVT, intravenous thrombolysis; MTE, mechanical thrombectomy

A comparison of patient characteristics for the years 2019 and 2021 can be found in Table 2. Overall IS patients and those who received IVT were more frequently male, whereas patients that received MTE were more frequently female, both in 2019 and 2021 (53.5% in 2021). A substantial increase of IVT rates was seen from 2016 to 2019 from 14.4 to 16.3%. This increase slowed after 2019 and IVT rates remained stable with 16.3% of patients receiving IVT both in 2019 and 2021 and 16.1% receiving IVT in 2022 (see Fig. 1).

**Table 2:** Patient characteristics and stroke treatments for 2019 and 2021 (Data from the Destatis database)

Variable	2019	2021	Test	*p* value
IS, n	225,531	217,002		
Age (mean ± SD)*	74.2 ± 4.9	74.2 ± 4.8	T test	*p* = 1.000
Sex, male, n (%)*	118,755 (52.7%)	115,648 (53.3%)	χ^2^ test	*p* < 0.001
≥ 80 years, n (%)*	88,332 (39.2%)	88,066 (40.6%)	χ^2^ test	*p* < 0.001
IVT rate, n (%)	37,009 (16.4%)	35,831 (16.5%)	χ^2^ test	*p* = 0.360
Age (mean ± SD)**	73.6 ± 4.6	73.5 ± 4.6	T test	*p* = 0.003
Sex, male, n (%)**	19,442 (52.5%)	19,076 (53.2%)	χ^2^ test	*p* = 0.056
≥ 80 years, n (%)**	14,062 (38.0%)	14,023 (39.1%)	χ^2^ test	*p* = 0.002
MTE rate, n (%)	16,135 (7.1%)	18,255 (8.4%)	χ^2^ test	*p* < 0.001
Age (mean ± SD)***	74.3 ± 5.0	74.4 ± 4.9	T test	*p* = 0.060
Sex, male, n (%)***	7331 (45.4%)	8497 (46.5%)	χ^2^ test	*p* = 0.039
≥ 80 years, n (%)***	6486 (40.2%)	7668 (42.0%)	χ^2^ test	*p* < 0.001
Number of regions with MTE rates ≤ 4.1%, n (%)	49 (12.3%)	21 (5.3%)	χ^2^ test	*p* < 0.001

*IVT* intravenous thrombolysis, *MTE* mechanical thrombectomy

* refers to all stroke patients, ** refers to all IVT patients, *** refers to all MTE patients

MTE rates increased substantially from 4.2% in 2016 to 7.1% in 2019 and continued to increase at a slower rate to 8.4% in 2021 and 8.7% in 2022. Overall MTE rates more than doubled from the year 2016 to 2022. This upward trend persisted throughout the years of the COVID pandemic. In 2022, a total of 18,809 MTEs were performed in comparison to 9795 in 2016.

**Fig. 2:**
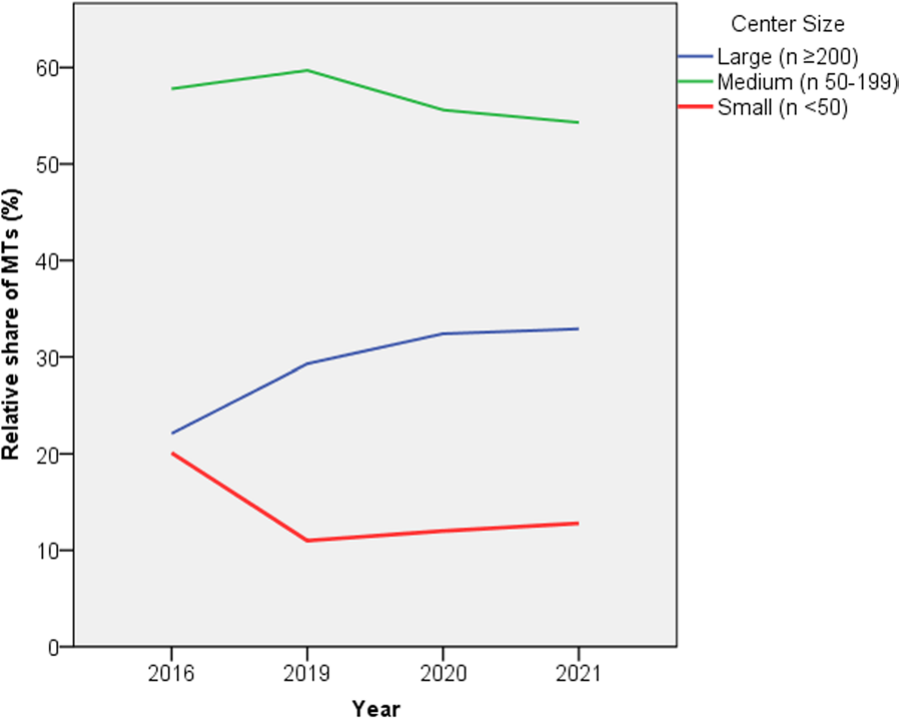
Relative share of MTEs performed per year in MTE centers according to case volume. Abbreviations: MTE, mechanical thrombectomy

**Fig. 3:**
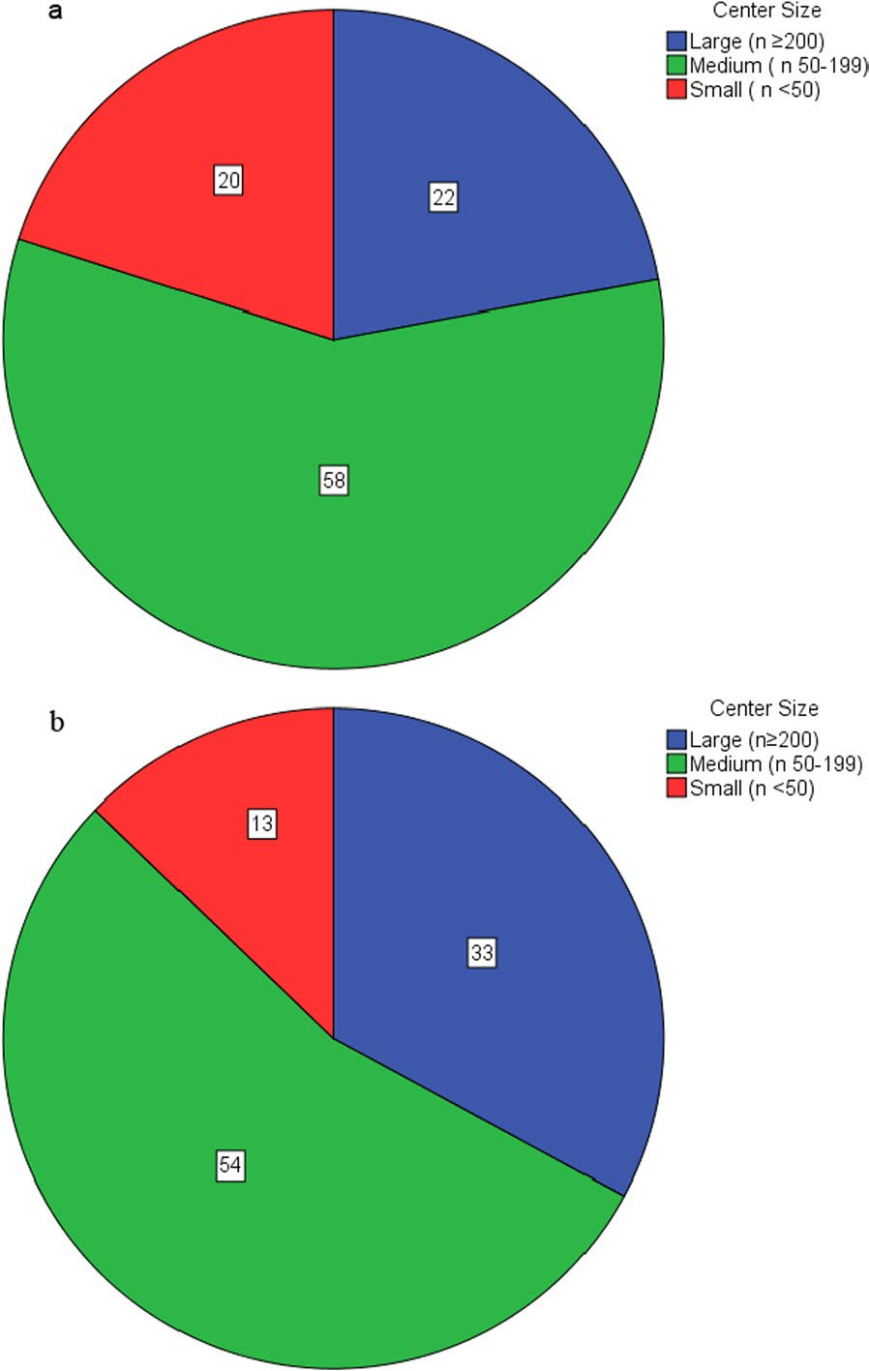
Relative share of MTEs (%) by center size in 2016 (**a**) and in 2021 (**b**). Abbreviations: MTE, mechanical thrombectomy

The number of centers performing MTEs increased by 41.5% from 159 in 2016 to 225 centers in 2021. The increase in MTE centers slowed after an initial rise by 23.3% (2016–2019) to 14.8% (2019–2021). The largest growth rate in both number of centers and share of MTEs in the period of 2019–2021 could be seen in large MTE centers (n ≥ 200) (see Figs. 2 and 3). The absolute number of large MTE centers increased by 144.4% from 9 to 22 centers with their relative share of MTEs increasing by 10.8% from 2016 to 2021 while the relative share of MTEs performed in medium (n 50–199) and small centers (n < 50) decreased by 3.4% and 7.4%, respectively. The absolute number of medium centers increased by 62.9%, while the number of small centers was relatively constant with an increase of 15.9% (2016–2021) to a total of 101 and 102 centers, respectively, in 2021.

7 MTE centers had a case volume of ≥ 300 per year in 2021 compared to just two in 2016. The majority of thrombectomies, accounting for 54.4% of MTEs in 2021, were performed in medium sized MTE centers. The great majority of MTEs in Germany were performed in centers with a case volume ≥ 50 MTE cases per year. This number increased from 79.9% in 2016 to 89.1% in 2019 and 87.3% in 2021. The relative share in MTEs of centers with less than 50 MTEs per year fell by almost half in the period from 2016 to 2019 and remained stable until 2021.

**Fig. 4:**
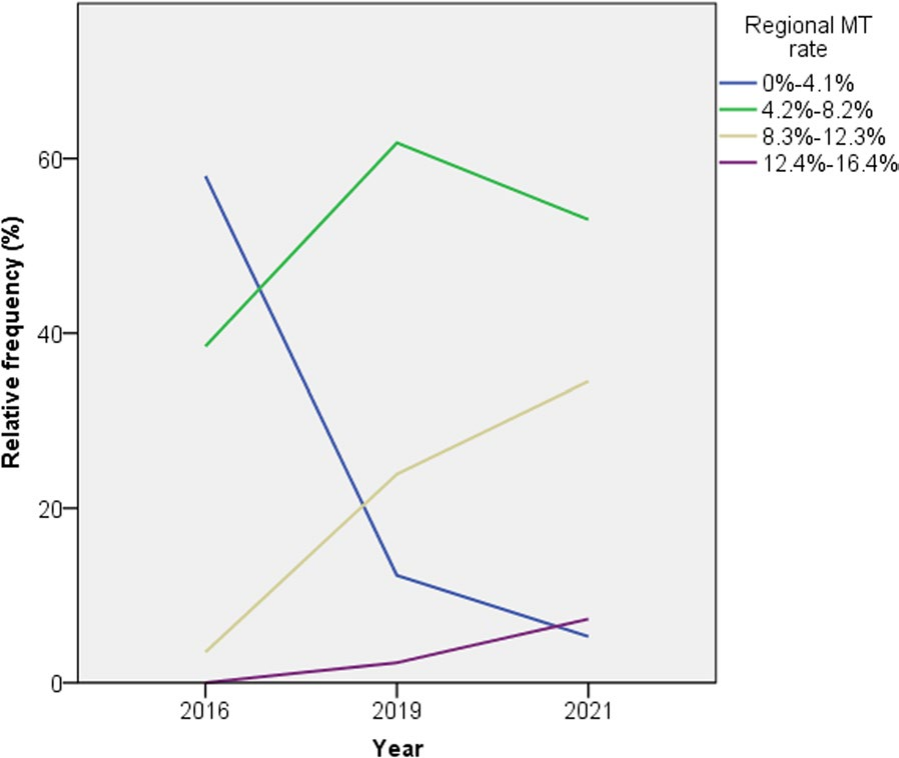
Relative frequency of regional MTE rates per year. Abbreviations: MTE, mechanical thrombectomy

Regional MTE rates increased substantially and became more evenly distributed from 2019 to 2021 (see Table 3). Relative frequency of regional MTE rates for 2016, 2019 and 2021 can be seen in Fig. 4. The relative share of regions with poor MTE rates (i.e. 0–4.1%) decreased substantially from 58.0% in 2016 to 12.3% in 2019 and 5.3% in 2021 (*p* < 0.001) indicating an increasingly even distribution of access to MTEs. Mean age for patients receiving MTE has also steadily increased and has even surpassed the mean age for all IS patients and for IVTs (*p* < 0.001) in 2019 and has remained higher throughout 2021. The relative MTE share for patients older than 80 years has increased from 32.4% in 2016 to 40.2% in 2019 and 42.0% in 2021. The relative share of MTE patients that were ≥80 years old surpassed the relative share of IVT patients that were ≥80 years old in 2019 and has since continued to increase.

**Table 3:** Frequency of regional MTE rates

MTE rate	2016	2019	2021
0–4.1% (n, %)	232 (58.0%)	49 (12.3%)	21 (5.3%)
4.2–8.2% (n, %)	154 (38.5%)	247 (61.8%)	212 (53.0%)
8.3–12.3% (n, %)	14 (3.5%)	95 (23.9%)	138 (34.5%)
12.4–16.4% (n, %)	0 (0%)	9 (2.3%)	29 (7.3%)

*MTE* mechanical thrombectomy

## Discussion

The main findings of our study can be summarized as follows: (1) MTE rates and absolute numbers continued to increase, although the rate of the increase has slowed substantially. We have also found evidence that access to MTE continued to increase both in terms of the geographical distribution of MTEs and in terms of access to MTE to patients ≥ 80 years old. (2) Both the relative increase in the number of MTE centers as well as MTE share was greatest in large MTE centers (n ≥ 200). (3) The majority of MTEs were performed in medium-sized MTE centers (n 50–199). Only 12.8% of all MTEs were performed in hospitals with less than 50 cases per year. (4) IVT rates were not negatively affected by the COVID pandemic but appeared to have reached a ceiling effect both in terms of regional distribution and relative rates while MTE rates continued to increase. The numbers of IS patients has not reached prepandemic levels as of 2022.

Ad (1) Our results suggest that MTE rates have grown substantially since the landmark trials were conducted in 2015 and have continued to increase steadily even 7 years after their publication. This is partly due to an increase in the numbers of MTE centers but also in the management of patients in community hospitals and prehospital management of stroke patients which encourage timely transfer and presentation of IS patients in MTE centers and increased awareness of physicians for this new treatment option. The continuous rise in MTE rates is also due to an expansion in the pool of eligible IS patients for MTE. In addition to the continuously expanding time-window for MTE and improvements in the availability of neuroimaging techniques (MRI, CT-perfusion) to identify eligible patients, new trials are studying the effect of MTE in previously ineligible subpopulations [10]. The fact that the relative MTE share for the elderly was high and kept on increasing suggests that MTE capacities continue to increase allowing MTE Centers to provide more MTEs to this population despite a more restrictive treatment approach in the early days of MTE. This is an indicator of greater equality of treatment. Because MTE is mostly available in larger hospitals due to the necessary infrastructure and skilled staff required for the performance of MTEs, disparities in access to MTEs remain a significant issue, even more than for IVT [1]. Particularly rural patients and patients living in underdeveloped regions have a lower MTE rate and worse outcomes after MTE in part because of longer transfer times [17, 21]. However we were able to demonstrate a continuing trend towards a reduction in the number of regions with poor access to MTEs in Germany. This is likely due to the successful implementation of drip and ship, mothership as well as ship the doc and FIT models which have contributed to an increased regional availability of MTEs. Particularly the establishment of drip and ship concepts have also contributed strongly to the increase in case volumes treated in large MTE centers.

Ad (2 and 3) Overall numbers of MTE centers of all sizes have increased since 2019, in line with findings from our regional MTE rate analysis supporting the fact that MTE coverage has become more widespread and evenly distributed as absolute case numbers have increased on a national level. Previous studies have suggested that low case volumes can negatively affect quality and outcomes of MTEs [6, 13, 22]. Our analysis of MTE shares according to center size has demonstrated that the relative number of thrombectomies performed at small MTE centers (n < 50) has remained largely unchanged despite an increase in the number of these centers. The largest relative increase was seen in large MTE centers (n ≥ 200) that are likely to provide high quality MTEs performed by experienced staff. This suggests that while regional access to MTEs increased across Germany from 2019 to 2021, treatment quality is unlikely to have been compromised in the process. Importantly, less than 12.8% of patients were treated in small MTE centers (n < 50) which would be considered to be “low volume” centers by international standards [25]. It is also likely that thrombectomies performed at small MTE centers are increasingly performed by mobile neurointerventional teams dispatched from larger MTE centers as part of ship the doc or FIT programs, which in part explains that the absolute number of small MTE centers has continued to increase. The large increase in medium-sized centers suggests that former small centers grew into medium-sized centers, indicating that MTE providers run through an implementation phase. Our data also showed that the medium sized stroke centers are the backbone of the MTE provision in Germany, as the majority of MTEs are performed at these centers. The increasing number of MTEs performed in large MTE centers in combination with increasing regional access to MTEs indicate a successful implementation of drip and ship concepts which expand the pool of MTE-eligible patients treated in large stroke centers. The effect of stroke and teleneurological networks as well as newly introduced concepts such as ship the doc and FIT on the improvement in regional availability of MTEs should be investigated in future research.

Ad (4) We found that the number of IS patients hospitalized in Germany during the COVID-19 pandemic fell substantially in comparison with pre-pandemic levels. This was described in Germany previously by Richter et al. [16] who compared IS numbers from 2020 and 2019. We were also able to confirm that IVT rates remained stable during this period and that MTE rates continued to increase. Similar declines in IS hospitalizations with rising MTE rates both in Germany and internationally in these years were described in several other publications [3, 12]. Interestingly, IS numbers in 2022 did not rebound to pre-pandemic levels despite most COVID restrictions and anxiety about the pandemic having abated in 2022. It remains to be seen whether this trend continues or whether IS numbers will increase again in the coming years to pre-pandemic levels. As to the IVT rate we found that a ceiling effect has been reached in Germany. However we cannot entirely exclude that the IVT rate has not been affected by the lower IS numbers as a result of the COVID-19 pandemic and that the rate has stagnated as a result of pandemic restrictions. It is also likely that changes in treatment paradigms, particularly the growing acceptance of IVT in patients on direct oral anticoagulants will lead to a renewed increase in the IVT rate in the coming years [14]. Future studies using large national datasets are necessary to study the effect of new guidelines and recommendations on this topic. We found a significantly increased relative frequency for patients receiving MTE to be female, while IS patients in general and those who received IVT were more frequently male. This is in line with the previous report by Richer et al. [18] and with other international studies which have reported higher female share among MTEs [4]. This is most likely because of sex differences in the etiology of stroke with females being more likely to suffer from embolic strokes and strokes of greater severity [5, 15].

### Strengths and limitations

The main strength of our study is the high completeness of a representative dataset that accurately depicts IS admissions, IVT and MTE rates throughout Germany. High completeness of data results from the necessity of hospitals to declare IS diagnoses and OPS procedures for reimbursement meaning that hospitals have a high incentive to document cases accurately.

Our analysis is limited by the kind of data that was available in association with diagnosis and coded procedures. We did not have access to data on patient outcomes or specifics on the MTEs performed (kind of vessel occlusion, method of MTE used etc.) allowing for only general statements to be made on the state and trends of MTEs in Germany. Data was extracted from different databases resulting in small differences in the numbers for MTEs and IVTs obtained. Data on case volume for individual MTE centers revealed slightly higher frequencies for IVT and MTE than the data retrieved from the destatis and InEK databases since MTEs and IVT procedures in combination with in-hospital strokes and double inclusions could not be excluded in these data. However all data used were checked thoroughly for plausibility and we provided most of the raw data as Additional file for transparency (see Additional File 1).

## Conclusions

The number of mechanical thrombectomies in stroke patients continued to increase in the period of 2019–2021 while rates for IVT reached a temporary ceiling effect. Access to mechanical thrombectomy continued to increase both for vulnerable patient subgroups and in terms of regional access. The largest growth in the relative share of MTEs was seen in large MTE centers which contributed strongly to a reduction in regional disparities in access to MTE presumably through drip and ship concepts. Further studies should determine the effect of mobile neurointerventionalist teams from large MTE centers on the reduction of regional disparities. The majority of MTEs were performed in medium sized MTE centers that provide the backbone of MTE treatment in Germany. Medium sized MTE centers should therefore receive greater consideration in future stroke treatment concepts.

### Supplementary Information

**Additional file 1**. Source data.

## Data Availability

Data analysed in this study are included in this published article [and its supplementary information files].The original datasets used and/or analysed during the current study are available from the corresponding author on reasonable request.

## Abbreviations

G-DRG: German-diagnosis related groups
FIT: Flying intervention team
IS: Ischemic stroke
IVT: Intravenous thrombolysis
MTE: Mechanical thrombectomy
OPS: Operationen- und Prozedurenschlüssel
SU: Stroke unit
