# Supplementary material for: The evolution of acute stroke care in Germany from 2019 to 2021: analysis of nation-wide administrative datasets
Source: Neurol Res Pract. 2024 Jan 11;6:4. doi: 10.1186/s42466-023-00297-x (PMC10782681; doi:10.1186/s42466-023-00297-x)
Supplement: Supplementary file 1 — Additional file 1. Source data. [file 42466_2023_297_MOESM1_ESM.docx]

**Supplemental materials: The evolution of acute stroke care in Germany from 2019 to 2021: Analysis of nation-wide administrative datasets**

Table 1: Absolute and relative frequencies for MTs, IVTs and IS admission in Germany

|  | 2016 | 2019 | 2020 | 2021 | 2022 |
| --- | --- | --- | --- | --- | --- |
| IVT-Rate | 14.4% | 16.3% | 16.4% | 16.3% | 16.1% |
| MT-Rate | 4.2% | 7.1% | 7.8% | 8.4% | 8.7% |
| I63 (after exclusion of “discharge key 6”) | 234,587 | 227,258 | 212,926 | 216,923 | 215,479 |
| I63+IVT | 33,823 | 37,009 | 34,896 | 35,363 | 34,719 |
| I63+MT | 9,795 | 16,135 | 16,701 | 18,192 | 18,809 |

Table 2a: Number of centers performing MTs according to case volume per year (Center Size according to original source data)

| Center Size | 2016 | 2019 | 2020 | 2021 |
| --- | --- | --- | --- | --- |
| n≥300 | 2 | 6 | 9 | 7 |
| n 200-299 | 7 | 11 | 11 | 15 |
| n 100-199 | 27 | 55 | 46 | 54 |
| n 50-99 | 35 | 44 | 53 | 47 |
| n 20-49 | 52 | 45 | 55 | 63 |
| n <20 | 36 | 35 | 32 | 39 |
|  | 159 | 196 | 206 | 225 |

Table 2b: Number of centers performing MTs according to case volume per year (Center Size as used in the manuscript)

| Center Size | 2016 | 2019 | 2020 | 2021 |
| --- | --- | --- | --- | --- |
| n≥200 | 9 | 17 | 20 | 22 |
| n 50-199 | 62 | 99 | 99 | 101 |
| n <50 | 88 | 80 | 87 | 102 |
|  | 159 | 196 | 206 | 225 |

Table 3a: MT rates according to case volume per year of MT centers (Center Size according to original source data)

| Center Size | 2016 | 2019 | 2020 | 2021 |
| --- | --- | --- | --- | --- |
| n≥300 | 7.2% | 15.3% | 19.0% | 15.2% |
| n 200-299 | 14.9% | 14.0% | 13.4% | 17.7% |
| n 100-199 | 33.8% | 42.7% | 36.2% | 37.7% |
| n 50-99 | 24.0% | 17.1% | 19.4% | 16.7% |
| n 20-49 | 16.2% | 8.6% | 10.1% | 10.6% |
| n <20 | 3.9% | 2.4% | 1.9% | 2.1% |
|  | 100.0% | 100.0% | 100.0% | 100.0% |

Table 3b: MT rates according to case volume per year of MT centers (Center Size as used in the manuscript)

| Center Size | 2016 | 2019 | 2020 | 2021 |
| --- | --- | --- | --- | --- |
| n≥200 | 22.1% | 29.3% | 32.4% | 32.9% |
| n 50-199 | 57.8% | 59.8% | 55.6% | 54.4% |
| n <50 | 20.1% | 11.0% | 12.0% | 12.7% |
|  | 100% | 100% | 100% | 100% |

Table 4: Frequencies for IVT, MT and IS admissions according to patient age

|  |  | Age group | | | | | | | | |
| --- | --- | --- | --- | --- | --- | --- | --- | --- | --- | --- |
|  | Total cases | <20 | 20-30 | 30-40 | 40-50 | 50-60 | 60-70 | 70-80 | 80-90 | >90 |
| IVT in 2016 | 33,918 | 42 | 128 | 444 | 1,516 | 3,570 | 5,751 | 10,409 | 9,630 | 2,428 |
| IVT in 2019 | 37,009 | 23 | 168 | 538 | 1,407 | 4,095 | 6,657 | 10,059 | 11,248 | 2,814 |
| IVT in 2021 | 35,831 | 26 | 133 | 524 | 1,362 | 3,970 | 6,759 | 9,034 | 11,246 | 2,777 |
| MT in 2016 | 9,794 | 13 | 55 | 134 | 459 | 1,076 | 1,711 | 3,167 | 2,732 | 447 |
| MT in 2019 | 16,135 | 19 | 69 | 167 | 540 | 1,635 | 2,736 | 4,483 | 5,358 | 1,128 |
| MT in 2021 | 18,255 | 24 | 63 | 239 | 599 | 1,811 | 3,265 | 4,586 | 6,279 | 1,389 |
| IS in 2016 | 227,688 | 275 | 747 | 2,308 | 8,145 | 23,400 | 39,218 | 69,993 | 67,184 | 16,418 |
| IS in 2019 | 225,531 | 253 | 724 | 2,372 | 7,025 | 22,998 | 40,638 | 63,189 | 71,789 | 16,543 |
| IS in 2021 | 217,002 | 235 | 732 | 2,385 | 6,752 | 22,126 | 40,523 | 56,183 | 71,851 | 16,215 |
